# Combating Stunting in Children Under Five Years Old: A Narrative Review of Policies and Programs in Southeast Asia

**DOI:** 10.7759/cureus.100127

**Published:** 2025-12-26

**Authors:** Siti Nurhuda Maisa, Hasliza Abu Seman, Nurul Balquis Azlan, Syaza Lyana Idris, Nur Amalina Amirullah, Norhasmah Sulaiman

**Affiliations:** 1 Department of Nutrition, Universiti Putra Malaysia, Serdang, MYS; 2 Undernutrition Research Program, Universiti Putra Malaysia, Serdang, MYS

**Keywords:** health policies, malnutrition, social determinants of health, southeast asia (sea), stunting

## Abstract

Southeast Asia is still battling with stunting cases, which remain a health challenge as it negatively hinders child development as well as impact national economic growth. Stunting results from inadequate nutrition and frequent infections during the first 1,000 days of life. This condition not only affects physical growth but also cognitive development, academic achievement, and later-life health, perpetuating a cycle of malnutrition across generations. Several countries in the region have implemented policies and programs to tackle this problem, focusing on both nutrition-specific and nutrition-sensitive interventions. These interventions vary from community-based initiatives, breastfeeding promotion, to comprehensive national nutrition plans. Despite these efforts, challenges remain, including socioeconomic disparities and the need for better coordination and monitoring of interventions. This study reviews the prevalence of stunting in Southeast Asia and examines the existing policies and programs aimed at reducing stunting rates in the region. This narrative review synthesizes current evidence on stunting in Southeast Asia to clarify regional gaps and guide targeted interventions. It provides a practical reference for strengthening policies and programs aimed at reducing stunting.

## Introduction and background

Malnutrition is a significant health issue affecting children’s development in many nations across the world. According to a World Health Organization (WHO) 2022 report, 148.1 million children under the age of five are too short for their age (stunting), 45.0 million are too thin for their height (wasting), and 37.0 million are too obese for their height (overweight). According to WHO standards, stunting is defined as having a height-for-age z score (HAZ) of less than -2 standard deviation (SD). It is calculated by taking a standard population, subtracting an age- and sex-appropriate median value from it, and then dividing the result by the standard population’s SD [1]. When fewer than 2.5% of children have HAZ below -2 SD, it suggests the population is generally healthy. However, if more than 2.5% of children fall below -2 SD, it indicates the children are living in a more disadvantaged or unhealthy environment [2]. Alarmingly, the prevalence of child stunting in Southeast Asia is at 27.4% in 2020 [3]. According to data published by Adara Relief International (2023), there are still countries in Southeast Asia where the prevalence of stunting is beyond the tolerance limit [4].

Child health is one of the indicators used by governments to assess the success of a nation in preparing the next generation. The WHO set the estimated limits of 20% stunting cases per country. If efforts to treat and prevent stunting remain ineffective, stunting is likely to worsen poverty and inequality if not properly addressed. International evidence demonstrates that stunting can also impact economic growth and diminish labor market productivity, leading to a loss of 11% of the GDP and a 20% reduction in worker maturity income [5].

Stunting that is caused by malnutrition in early life has negative lasting effects, both physically and psychologically, as a result of insufficient nutrition and frequent infections during a child’s first 1,000 days of life. Stunting can affect a child’s development and has lasting effects into adulthood [6]. First, stunting causes damage that can irreparably affect long-term health and development. Early growth failure has been associated with increased infection risk, delayed cognitive development, and worse academic achievement in childhood and adolescence [7]. Second, stunting significantly affects adult height. In women, this influences both the health and survival of their children as well as their own reproductive health. In men, reduced height is linked to decreased economic productivity [7]. Stunting can also lead to obesity, which increases the risk of non-communicable diseases (NCDs) by placing a high metabolic load on a body that has lost its ability to maintain homeostasis [8]. Moreover, women of shorter stature face higher risks of obstetric complications due to their smaller pelvis size. It results in an increased likelihood of delivering infants with low birth weight, perpetuating a cycle of malnutrition where low birth weight infants are more likely to remain small as adults. This reveals a further amplification of the triple burden of malnutrition, which can result in a cycle that lasts many generations to come [9].

Sociodemographic conditions such as household poverty, caregiver education, and access to safe water and sanitation shape food security, caregiving practices, and children’s exposure to infection, which, in turn, influence dietary intake, morbidity, and linear growth and thereby the risk of stunting. National policies across sectors (health, social protection, water, sanitation, and hygiene (WASH), agriculture, and education) are designed to act on these upstream determinants by improving service access, reducing poverty and vulnerability, and strengthening caregiver knowledge and resources, with the ultimate goal of creating healthier environments and behaviors that lead to lower stunting prevalence and better child growth outcomes [10,11].

This narrative review aims to identify the prevalence of stunting in Southeast Asian (SEA) countries and to examine the policies and programs implemented across SEA countries to address stunting, as many of the key determinants of stunting, such as poverty, parental education, sanitation, and access to healthcare, are largely sociodemographic in nature. These factors often extend beyond individual shortcomings or household-level choices, highlighting the need for systemic, policy-driven approaches. This review also analyzes the latest stunting prevalence in each country, comparing it with the targets set in policy documents. By analyzing how different nations in the region are responding, this review aims to identify strengths, gaps, and opportunities for more effective strategies to break the cycle of stunting.

## Review

Methodology

Policies targeting the treatment of stunting through the promotion of healthy lifestyles and improved nutrition among infants and young children were initially identified through comprehensive electronic searches of authoritative organizational websites. These included the WHO (https://www.who.int/), the Centers for Disease Control and Prevention (CDC) (https://www.cdc.gov/), and the United Nations Children’s Fund (UNICEF) (https://www.unicef.org/), which serve as primary global reference points for child health and nutrition policy frameworks.

To ensure the inclusion of high-quality, valuable, and evidence-based literature, a further search was conducted in electronic databases, including Google Scholar, PubMed, and ScienceDirect. Any relevant government agency platforms were also included to identify articles on stunting policies in SEA. Policy documents of each country on maternal and child health and nutrition were included, if the English version was available. If multiple versions were available, the most recent ones were reviewed. The search was conducted using the keywords “stunting,” “policy,” “nutrition,” “southeast asia,” and “children.” Reviews and guidelines were evaluated for their applicability, with the goal of adding new materials to the review. Articles that focused either fully or partially on promoting good nutrition for infants and children and were published in the English language by endorsed organizations (such as WHO, UNICEF, or other organizations) were eligible for our study. These articles included reports, action plans, and policy briefs. These documents were included in the review as they are the source of several national policies in SEA countries, with government publications deemed relevant to this work. Publications about nutritional treatments or policy implementation, as well as those that only addressed goals related to adolescents, or mentioned no policies specifically targeted at children, were excluded from the analysis. There were no limitations on the date of publishing. The search for literature lasted until June 2024. Key information extracted from each source included policy name, time period, and outcome indicators.

Prevalence of stunting in children under five: globally and in Southeast Asia

**Figure 1:**
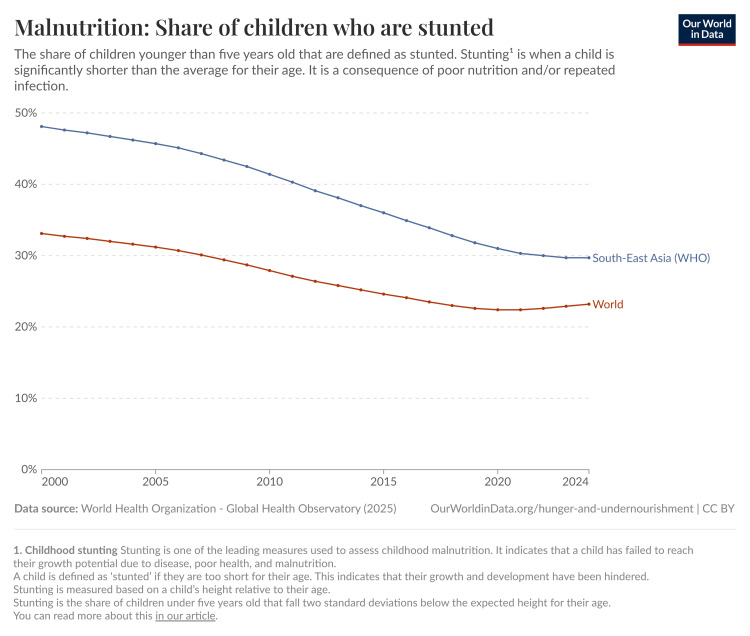
Prevalence of stunting globally and in Southeast Asia from 2000 to 2024. Figure reproduced from Our World in Data (2024) under the Creative Commons License (CC BY) [13].

In 2012, the World Health Assembly (WHA) designated stunting as one of the six key areas in the Global Nutrition Targets. The aim was to enhance maternal, infant, and young child nutrition by 2025, with a goal of achieving a 40% reduction in the number of children under five affected by stunting [12]. Since 2000, there has been a gradual decline in global stunting prevalence among children under five years old. Recent estimates indicate that approximately 22.0% of children worldwide were stunted in 2020, down from about 33.1% in 2000 (Figure 1). In Southeast Asia, stunting among children under five years old remains a significant challenge, with an average prevalence of 27.4% in 2020, down from 38.0% in 2000, but still higher than the global average (Figure 1) [13].

**Figure 2:**
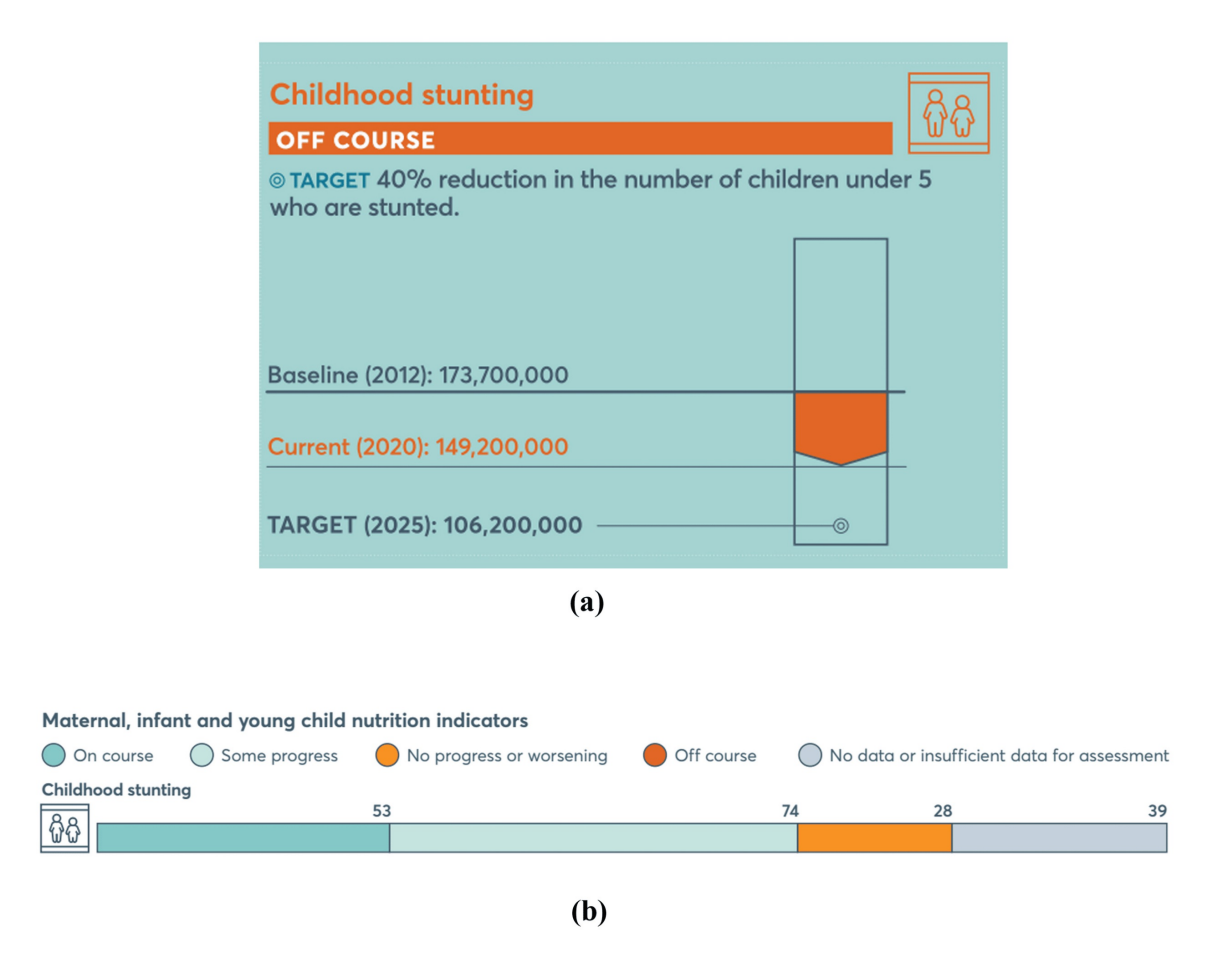
(a) Global progress toward 40% reduction in the number of children under five years old who are stunted. (b) Country-level progress toward the reduction of childhood stunting set by the Global Nutrition Target 2025. Figure adapted from the Global Nutrition Report (2022), which is licensed under a Creative Commons Attribution BY-NC-ND 4.0 International license [14].

**Figure 3:**
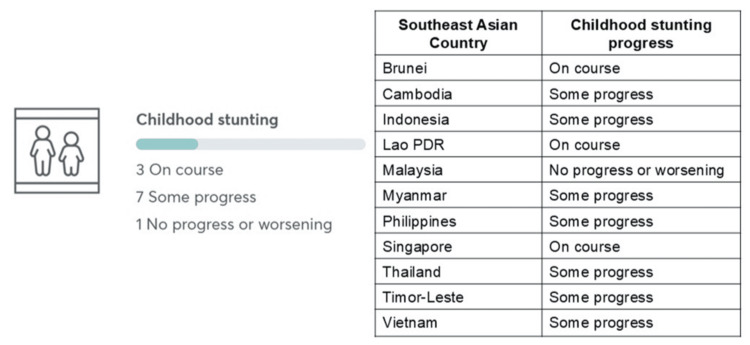
Progress towards reduction of childhood stunting in Southeast Asia regions set by the Global Nutrition Target 2025. Figure adapted from Global Nutrition Report (2022), which is licensed under a Creative Commons Attribution BY-NC-ND 4.0 International license [15].

As of 2022, 148.1 million children under five years were stunted globally, marking a decline from previous years (Figure 2a) [14]. However, the rate of progress remains insufficient, with only about one-third of countries on track to meet the 2025 target (Figure 2b). Significant regional disparities exist, with the majority of stunted children residing in Asia and Africa. In the Southeast Asia region, only three countries are on track to achieve the childhood stunting target, while seven countries show only partial progress (Figure 3) [15]. Additionally, one country shows no progress or worsening in addressing childhood stunting.

**Figure 4:**
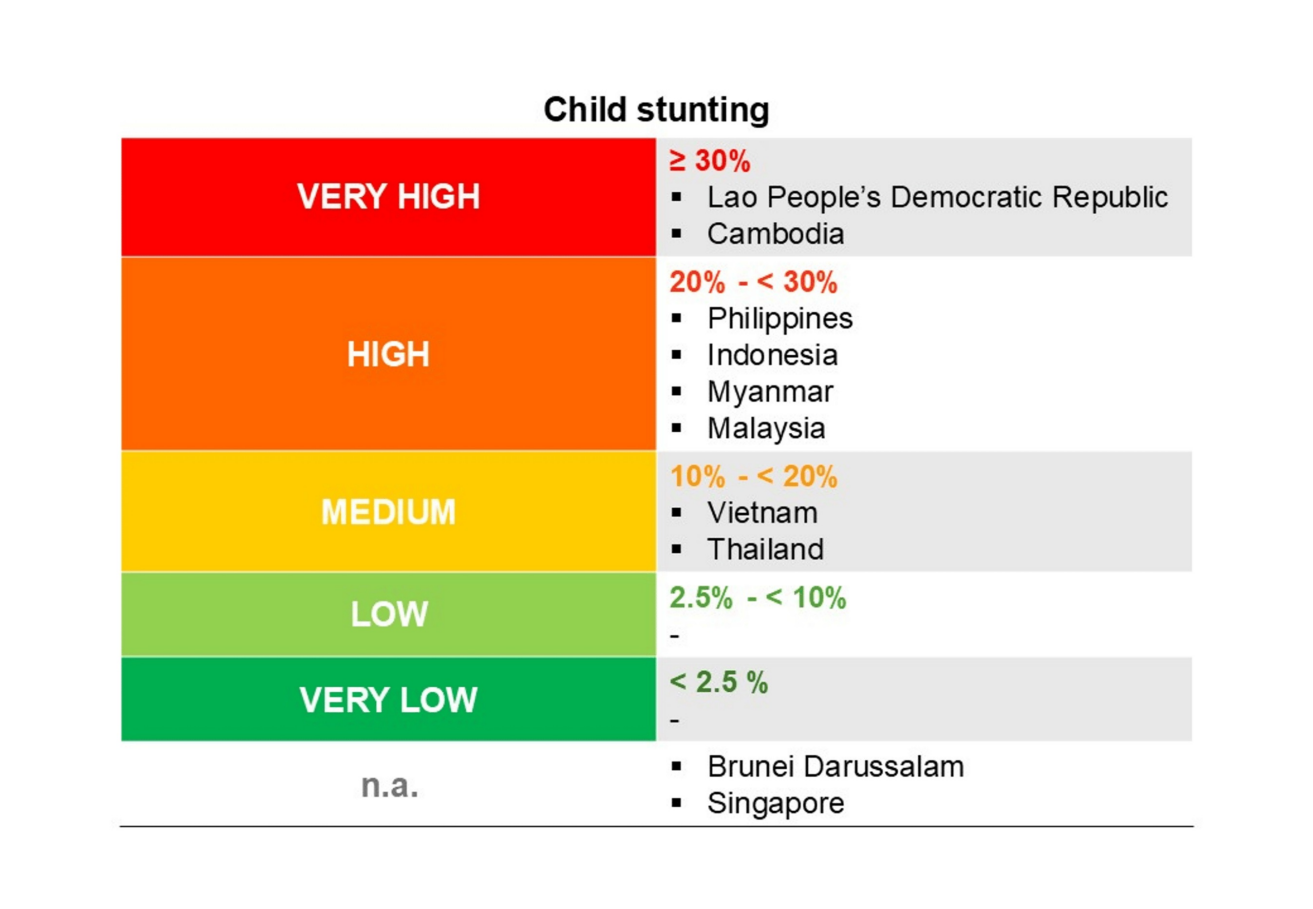
Risk levels of stunting. Figure adapted from ASEAN Food and Nutrition Security Report (2021) under the Creative Commons License (CC BY) [16].

**Figure 5:**
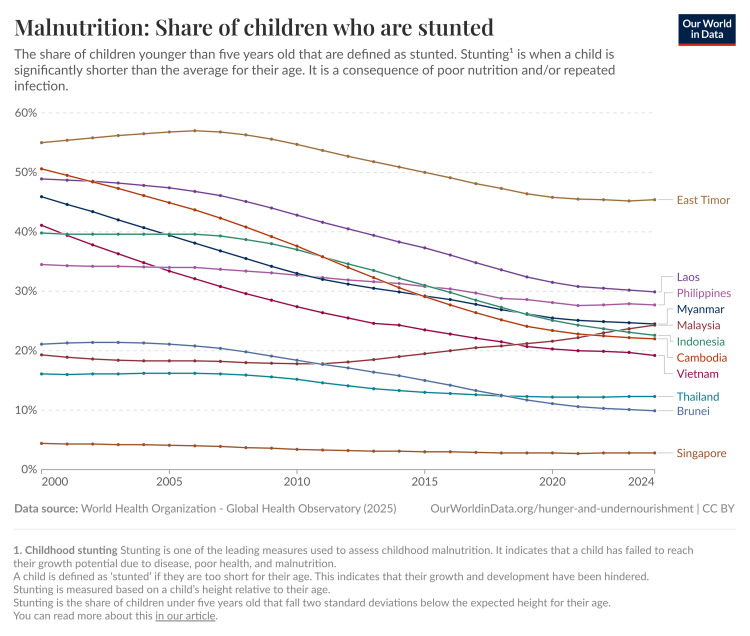
Stunting prevalence in Southeast Asian countries. Figure reproduced from Our World in Data (2024) under the Creative Commons License (CC BY) [17].

Despite significant reductions since 1990, stunting remains a critical issue in Southeast Asia, affecting 15 million children under five. According to the ASEAN Food and Nutrition Security Report 2021, Cambodia and the Lao People’s Democratic Republic (PDR) are classified as having “very high” stunting prevalence (over 30%), while Indonesia, Malaysia, Myanmar, and the Philippines fall under the “high” category (20% to 30%). Thailand and Vietnam are categorized with “medium” stunting prevalence (10% to 20%), highlighting ongoing challenges in combating this issue across the region. This data excludes Singapore and Brunei (Figure 4). Stunting is uncommon in Singapore, and therefore, national statistics on the condition are not tracked. Data from Brunei Darussalam were collected before 2010, and therefore, were not included in this report [16]. Stunting trends from 1987 until 2020 are presented in Figure 5 [17]. Inequalities in the burden of malnutrition and diet quality among the various population groups in the ASEAN region are revealed by a deeper investigation of malnutrition data. For example, children from the poorest homes are more likely than those from wealthier households to suffer from stunting and wasting. This is valid for the ASEAN member states as well as for the world at large. When comparing children living in rural versus urban environments, the prevalence of stunting and wasting is often higher in the former [18]. It is noteworthy, therefore, that large-scale household surveys frequently omit the urban poor. There is proof that the frequency of malnutrition varies greatly throughout metropolitan locations in the ASEAN region, with the poorest households more at risk for stunting.

Discussion

The WHO Stunting Policy Brief (2014) recommended the implementation of affordable treatment and policies targeting to reduce stunting cases among children under five years of age, aligning with the Global Nutrition Target 2025 [19]. The new 2025 “Global nutrition targets 2030: Stunting brief” reiterates the global stunting target but updates the epidemiology, economic case, and recommended actions. The 2025 stunting brief reports that global child stunting has declined from 26.4% in 2012 to 23.2% in 2024. However, this progress remains far from the original goal of a 40% reduction by 2025. As a result, the WHA has extended the same 40% reduction target to 2030 and called for intensified multisector investments and coordinated life-course interventions aligned with the updated WHO/UNICEF guidance and new initiatives [20].

Key strategies to combat stunting include setting national targets to strengthen the detection and assessment of stunting, alongside the integration of routine child growth monitoring within health services. Equally important is improving maternal health and nutrition by ensuring adherence to the code of marketing of breast-milk substitutes, promoting the use of iron and folate supplements, supporting safe birth practices, controlling infections, and encouraging exclusive breastfeeding. Furthermore, there is an emphasis on effective breastfeeding and complementary feeding practices, particularly through educating caregivers on the importance of nutrient-rich foods once exclusive breastfeeding ends at six months. Addressing environmental factors is also critical, with a focus on improving WASH as an underlying determinant of stunting. By integrating health services, nutrition education, and supportive policies, these recommendations aim to effectively combat stunting and improve child health outcomes at the global scale.

Policies and programs addressing stunting in Southeast Asian countries

The UNICEF Conceptual Framework on Maternal and Child Nutrition (2021) offers a comprehensive, multi-level view of the factors shaping maternal and child nutrition. It differentiates enabling determinants, such as governance, resource allocation, and social norms; underlying determinants, including the availability of nutritious foods, age-appropriate feeding and dietary practices, and access to nutrition, health, and sanitation services; and immediate determinants, which relate directly to the adequacy of diets and the quality of care received by women and children [11].

By incorporating stunting reduction goals into national nutrition strategies, governments recognize their significant impact on child development through dietary habits. This integration is crucial for identifying challenges and efficiently allocating resources to combat stunting. Without effective monitoring of stunting trends, governments risk lacking essential data needed to prioritize interventions or justify investments in nutrition initiatives. Moreover, without mechanisms for accountability, sustained attention, and funding for efforts to reduce stunting and enhance dietary practices may diminish over time. The following subsections discuss 10 SEA countries that have integrated targets for reducing child stunting into their national policies to align with global objectives. It should be noted that Singapore is an SEA country that has not included stunting in its national policies and does not monitor it due to low prevalence rates. The following discussion focuses on the policies in each country, especially on how they fit the UNICEF Conceptual Framework, particularly the enabling determinants (governance, resources, and norms).

Brunei

The National Strategy for Maternal, Infant, and Young Child Nutrition (MIYCN) 2014-2020 aimed to address the double burden of malnutrition (over- and undernutrition) for children aged zero to five years and women of reproductive age in Brunei Darussalam. It focused on enhancing the health and well-being of infants, young children, and mothers [21]. The National Strategy for MIYCN identifies six global targets, some of which include reducing the prevalence of anemia among women of reproductive age by 50% by 2025 and decreasing low birth weight by 30% within the same timeframe. By 2025, the policy aims for a 40% reduction in the prevalence of childhood stunting, and the rate of exclusive breastfeeding during the first six months of life is aimed to increase to at least 50%.

Additionally, the National Strategy for MIYCN also outlines several steps or goals to improve maternal and child health. There are discussions about making antenatal and postnatal services available and accessible, as well as the provision of supplements for pregnant women. Specifically, prophylaxis with folic acid and prophylaxis with ferrous fumarate supplementation for pregnant mothers. To encourage exclusive breastfeeding, there are several steps taken by the Brunei government, as mentioned in the National Strategy for MIYCN, such as expanding the Baby-Friendly Hospital Initiative (BFHI), placing the National Breastfeeding Policy, annual highlights of World Breastfeeding Week, and media promotional activities. There are also plans to expand the existing immunization programs for children.

Interestingly, the National Strategy for MIYCN also specifically mentions the Maternity Leave Regulation 2011. This regulation extended maternity leave from 56 to 105 days for married female citizens, permanent residents, and civil servants, with the aim of supporting exclusive breastfeeding among working mothers in the country [22]. Meanwhile, the National Health Promotion Blueprint 2011-2015 outlined strategic objectives and actions aimed at preventing and controlling NCDs while promoting healthier lifestyles nationwide [23]. It also called for the revision of the Infant and Young Child Nutrition Program to better address maternal nutrition concerns.

In 2010, Brunei achieved significant improvements in key health indicators, with under-five mortality reduced to 7.3 per 1,000 live births, infant mortality decreased to 6.1 per 1,000 live births, and maternal mortality lowered to 15.6 per 100,000 live births. Notably, the maternal mortality rate is comparable to that reported in developed countries [21].

Meanwhile, a recent WHO data estimated the stunting prevalence for children under five in Brunei to be 10.9% in 2022 [24]. Brunei’s maternal and child health performance is strong on most core indicators, with outcomes comparable to many high‑income settings. Maternal mortality is very low (around 16-44 deaths per 100,000 live births in recent estimates), under‑five mortality is under 10 deaths per 1,000 live births, and neonatal mortality is about 5 per 1,000, while ANC (≥4 visits) and skilled birth attendance are essentially universal, and childhood immunization coverage has long exceeded 95%. These results reflect long‑standing public investment in free, high‑quality primary care, an extensive maternal and child health clinic network, near‑universal facility delivery, strong childhood immunization and MIYCN strategies, and relatively high living standards [25].

Cambodia

Cambodia has multiple policies and programs to improve the health of its populace, such as the Fast Track Road Map for Improving Nutrition 2014-2020 [26] and the 2nd National Strategy for Food Security and Nutrition 2019-2023 [27].

The Fast Track Road Map for Improving Nutrition 2014-2020 focuses on enhancing maternal and child nutrition to reduce undernutrition in this vulnerable population [26]. The Nutrition Road Map outlines a strategic approach to achieve two main outcomes: scaling up nutrition-specific interventions to improve maternal and child nutritional status and removing barriers that hinder the effective implementation of these services. The plan includes eight major components: promoting nutrition counseling during ANC, sustaining and enhancing micronutrient supplementation for pregnant and lactating women (including daily iron and folic acid supplements), expanding nationwide management of severe acute malnutrition in children, scaling up the distribution of micronutrient supplements for young children, improving behavior change communication related to the first 1,000 days of life, addressing financial and human resource challenges to scaling up nutrition interventions, leveraging support from other ministries and initiatives, and enhancing nutrition data through existing information systems.

Meanwhile, the National Strategy for Food Security and Nutrition (2014) has three main objectives [28]. Objective 1 focuses on increasing food availability and access for food-insecure households through productive agriculture, sustainable fisheries and forestry, and non-agricultural income opportunities. This includes intensifying smallholder farming, improving market linkages, securing land access, and enhancing utilization of common property resources. Objective 2 aims to reduce child and maternal malnutrition and promote human and economic development by scaling up nutrition services, improving water supply and sanitation, fortifying food with micronutrients (such as iodized salt and iron-fortified fish and soy sauces), promoting nutritious and safe food at the household level, and integrating nutrition into social protection programs. It also includes piloting a community-based nutrition program in areas with high child malnutrition. Objective 3 focuses on enhancing food security-related social protection and building resilience among vulnerable households by scaling up social protection instruments, improving disaster preparedness and mitigation, and increasing household resilience against climate change impacts.

According to the 2nd National Strategy for Food Security and Nutrition (2nd NSFSN) 2019-2023, Cambodia’s national data from 2014 indicated that significant challenges remain, with 32% of children under five experiencing stunting, 24% being underweight, and 10% being wasted [27]. The 2nd NSFSN marks a significant shift in Cambodia’s approach by addressing both undernutrition and the challenges of overweight and obesity within its framework. The national strategy sets ambitious targets for improving the nutritional status of mothers. The goal is to decrease the prevalence of underweight (BMI <18.5 kg/m²) among women of reproductive age from 14% in 2014 to 10% by 2023. Additionally, efforts are focused on reducing anemia among women aged 15 to 49 years. To further support maternal health, the strategy includes scaling up maternal nutrition services and counseling, particularly during the critical 1,000-day window that encompasses pregnancy and early childhood. There is also a commitment to lower the prevalence of newborns with low birth weight from 8% in 2014 to 6% in 2023. To encourage breastfeeding among working mothers, the 2nd NSFSN also mentions the strengthening of the Law on Labor 1997, with particular emphasis on Articles 182 to 187 regarding maternity leave.

For children, the 2nd NSFSN aims to reduce stunting among those under five from 32% in 2014 to 25% in 2023, acknowledging that Cambodia continues to face a high prevalence of stunting. The plan also seeks to decrease wasting in children under five from 10% in 2014 to 8% in 2023. Furthermore, promoting optimal infant feeding practices is a priority, with a target to increase the rate of exclusive breastfeeding for infants aged zero to six months from 65% in 2014 to 68% by 2023. In addition, there is also a goal to improve Nutrition-Sensitive WASH, particularly in rural areas, as access to WASH in these areas still needs improvement.

A recent Cambodian Demographic and Health Survey (CDHS), 2021/2022, showed that 21.9% of children under five were stunted, 9.6% were wasted, and the rate of exclusive breastfeeding was 50% [29]. Overall, Cambodia seems to be on the right track to reduce the prevalence of stunting (although wasting prevalence and exclusive breastfeeding did not achieve the targets set in the 2nd NSFSN). Based on the achievements thus far, it might be prudent for existing policies and programs in Cambodia to remain or be improved based on the data obtained.

Indonesia

The Indonesian government has specific policies to address the issue of stunting in the country, such as the National Strategy to Accelerate Stunting Reduction (Stranas Stunting) 2018-2024 [30]. The overarching aim of Stranas Stunting is to expedite progress in reducing stunting within the existing policy and institutional frameworks. To achieve this, the strategy outlines five specific objectives: ensuring that stunting reduction is prioritized by both government and communities at all levels; raising public awareness and fostering community behavioral change to address stunting; enhancing program coordination and consolidation across central, regional, and village levels to strengthen convergence; improving access to nutritious food and promoting food security; and intensifying monitoring and evaluation efforts to guarantee quality service delivery, strengthen accountability, and facilitate accelerated learning. The Stranas Stunting document agrees that intensified measures are required to accelerate progress in reducing stunting. This is especially true to achieve the WHA target of reducing stunting prevalence by 40% from 2013 levels to reach 22% by 2025 [30].

The primary focus of the Stranas Stunting is on pregnant women and children aged 0-23 months, encompassing households during the crucial first 1,000 days of life. Key objectives include reducing the prevalence of anemia among pregnant women, with a baseline rate of 48.9%, and lowering the incidence of low birth weight, currently at 6.2%. The strategy also prioritizes improving maternal nutrition both before conception and throughout reproductive age.

In the Stranas Stunting, Indonesia’s nutrition and food security initiatives encompass a multifaceted approach, including the provision of iron and calcium supplements for pregnant mothers, as well as vitamin A and multiple micronutrient supplements for lactating mothers and young children aged 0-23 months. Disadvantaged families receive support through access to both cash assistance programs and non-cash food assistance. The country has improved access to fortified staple foods such as salt, wheat flour, and cooking oil. These strategies are facilitated by multi-stakeholder cooperation, involving the private sector, and are implemented at every administrative level from provincial to village. Indonesia has also integrated the 1989 Ten Steps to Successful Breastfeeding into national regulations, although there is currently no BFHI accreditation program in place [31]. While laws extending maternity leave exist, this measure does not appear to be included in the Stranas Stunting policy documents and may be found elsewhere.

The Presidential Regulation No. 18 of 2020 Concerning National Medium-Term Development Plan/RPJMN for 2020-2024 [32] mentions that the prevalence of stunting among children under five in Indonesia was 27.7% in 2019, and the prevalence is targeted to be reduced to 14% by 2024. Additionally, Indonesia’s involvement in the Scaling Up Nutrition (SUN) Movement shows its commitment to combat malnutrition [33]. The SUN Movement emphasizes the importance of cross-sectoral participation and social welfare policies in addressing stunting, aligning with the goals of the Indonesian government’s nutrition programs [34]. The SUN Movement provides a valuable framework for operationalizing stunting prevention activities, leveraging national frameworks and coordination mechanisms to drive progress in addressing malnutrition [35].

The Indonesian Nutritional Status Survey (SSGI) 2024 showed that the prevalence of stunting for children under five in Indonesia to be 19.8% [36]. While this did not reach the targeted 14% as in the Presidential Regulation No. 18 of 2020, it still proved that Indonesia has performed remarkably, despite all the challenges it faced. The success of these efforts depends on effective program design, coordination, and stakeholder engagement, emphasizing the importance of a holistic approach to nutrition interventions. Through ongoing research, evaluation, and adaptation, Indonesia can continue to refine its strategies for combating stunting and advancing public health outcomes.

Laos

The Lao PDR government has long committed to promote nutrition and eradicate all types of malnutrition, beginning with the first National Nutrition Plan (Prime Minister’s Decree 248) in 2008. This pledge has been renewed by the government in the new National Plan of Action on Nutrition (NPAN) 2021-2025 [37].

In 2017, Laos experienced substantial nutrition challenges, with 33% of children under five affected by stunting and 21% classified as underweight. The NPAN 2021-2025 is centered on maternal, infant, and young child nutrition, while also highlighting the interconnectedness of malnutrition with food security, food systems, agriculture, education, access to health services, primary healthcare, adolescent health, and disaster responses, including those related to climate change and pandemics such as COVID-19 [37]. The plan additionally stresses social and behavior change communication and gender equality. A multisectoral strategy to improving nutrition is considered fundamental in this NPAN, which includes an updated conceptual and strategic framework, featuring eight indicators for the overall goal, 13 strategic objectives, 22 interventions, and 36 outcome and output-level indicators.

By 2025, the NPAN aims to achieve several key health targets for mothers and children. For mothers, the prevalence of anemia among women of reproductive age is targeted to be 15%, and the rate of low birth weight is expected to decrease to 8%. The goals for children under five include reducing stunting to 26%, lowering the prevalence of wasting to less than 5%, and decreasing underweight rates to 13%. Additionally, the NPAN targets a 60% rate of exclusive breastfeeding among infants under six months by 2025. The national nutrition strategy emphasizes vitamin A supplementation for children under five and the provision of iron-folic acid tablets for pregnant women. There are overarching goals to strengthen the fortification of foods with vitamins and minerals, although no specific foods or micronutrients are named. The approach relies heavily on multisectoral cooperation, integrating efforts from sectors such as agriculture, health, and education.

In addition, there are goals to expand maternal, newborn, and child health (MNCH) services covering ANC, delivery care, and postnatal care, with a focused commitment to MIYCN during the first 1,000 days of life. Initiatives such as the BFHI and increased coverage of infant and young child feeding (IYCF) seek to improve early childhood outcomes. Broader support structures encompass school feeding programs, improvements in WASH systems, and safe water access. Social and behavioral communication campaigns aim to enhance community engagement, particularly among the poorest and most remote populations. Partnerships with organizations such as the World Food Programme (WFP), alongside social transfers and food assistance, are leveraged to build resilience and address food insecurity.

One example of a partnership is with the World Bank. This project focused on investing in 12 target districts, starting with Phongsaly, Oudomxay, Xieng Khouang, and Houaphanh, the four northern provinces having some of the highest rates of stunting in the nation [38]. With a focus on supporting the country’s multi-sectoral convergence approach to nutrition, its first step is to build and strengthen the institutional foundations of multi-sectoral interventions. The scope of the program is then increased in light of the information obtained about its operations. The Health and Nutrition Services Access (HANSA) Project is one of the initiatives, whereby several interventions were included such as providing pregnant women with iron-folic acid; providing vitamin A to children under five; monitoring children’s growth; implementing social and behavioral change communication (SBCC) through trained female village volunteers, enhancing water sources, supply systems, and sanitation; enhancing sanitation facilities and handwashing practices; and terminating open defecation.

Another program is the Accelerating Healthy Agriculture and Nutrition (AHAN) Project, led by World Vision and a consortium of partners [39]. The goal of this project was to address the underlying causes of malnutrition in rural Lao households with a high concentration of its efforts on mothers and children under five. The project was implemented between 2019 and 2021 with a total allocation of €11 million, primarily funded through the European Union’s Program for Improved Nutrition (EU PIN) and supported by Australia. The initiative operated in three provinces: Savannakhet, Salavanh (Saravane), and Attapeu, encompassing 12 districts and 120 villages (10 villages per district). In 2020, the AHAN project published qualitative research on the impact of feeding patterns on mother and child nutrition.

The recent Lao Social Indicator Survey III, 2023, Survey Findings Report showed that 32.8% of children under five were stunted, while 10.8% were severely stunted [40]. Laos has one of the highest stunting rates in Southeast Asia countries, because many children still face a combination of poor diets, unhealthy environments, and structural inequities that are only partially addressed by existing policies [41]. Diets in poorer and rural households are often low in diversity and animal-source or protein-rich foods. Meanwhile, families also frequently lack safe water, adequate sanitation, and good hygiene, leading to repeated infections and environmental enteric dysfunction that impair growth. These immediate and underlying causes are reinforced by persistent poverty, rural-urban and ethnic disparities, low maternal education, limited access to quality health and nutrition services, and implementation and coordination gaps in national nutrition plans, so progress in reducing stunting has been slower and more uneven than economic growth alone would suggest.

Malaysia

The National Plan of Action for Nutrition of Malaysia (NPANM) III 2016-2025 was comprehensively reviewed and reinforced to address rapid dietary changes and the dual burden of malnutrition [42]. The policy aims to enhance community well-being by improving nutritional status, combating nutrition-related NCDs, and bolstering food security through a sustainable food system. The NPANM III integrated nutrition into national policies, envisioning national nutritional wellness by reducing malnutrition burdens and enhancing food security. It contains four indicators, six enabling strategies, and five facilitating strategies. To facilitate these aims, the plan provides standard nutrition guidelines for targeted groups, ensures continuous assessment and monitoring of the nutrition situation, and promotes strengthened food and nutrition research and development.

Under enabling strategies, Malaysia’s nutrition and child health initiatives focused on several key areas. These included monitoring and educating about Gestational Weight Gain (GWG) based on IOM 2009 guidelines and enhancing nutrition services for economically disadvantaged pregnant women through the Urban Antenatal Nutrition Initiative (UANI B40) [43]. Additionally, updates to the Perinatal Care Manual (PCM) aimed to improve prenatal and postnatal care standards. The BFHI and Nursing Mothers Program promoted breastfeeding in hospitals and workplaces [44].

Under facilitating strategies, Malaysia’s initiatives related to nutrition and child health focus on policy and guideline alignment, monitoring and advocacy, training and capacity building, research and evaluation, and education and curriculum enhancement. These initiatives involve updating the Malaysian Dietary Guidelines (MDG) for 2020 and 2023 and incorporating them into pre-school nutrition and healthcare training [45,46]. Growth monitoring for children under five is emphasized, supported by the Malaysian Health Data Warehouse (MyHDW) for tracking progress [47]. There are also specific guidelines for the first 1,000 days of life launched by the Ministry of Health Malaysia [48].

A mid-term review of the NPANM III has targeted improvements for mothers and young children by 2025. For mothers, the goals are to reduce anemia during pregnancy to 19.5%, ensure half achieve healthy weight gain while pregnant, increase breastfeeding initiation to 90%, and lower low birth weight to under 9.2%. For children, the targets are to reduce stunting to 11.8%, wasting to 4.7%, and to keep underweight at or below 6.8%. Moreover, the rate of exclusive breastfeeding for babies under six months is set to reach 70% [44]. Despite these goals, the recent data from the National Health and Morbidity Survey (2022) showed that the prevalence of anemia during pregnancy among respondents aged 15-49 years was 19.3%, while 10.9% of infants were born with low birth weight. Among children under five, 21.2% were affected by stunting, 15.3% were underweight, and 11.0% experienced wasting [49].

Despite high coverage of ANC, facility delivery, and immunization, the structural drivers of malnutrition have not improved enough, and benefits are unevenly distributed across the population. National and local studies consistently show much higher stunting in poorer households, certain states (including parts of the East Coast and East Malaysia), and among Indigenous/Orang Asli children, indicating that strong services on paper are not reaching or adequately addressing the needs of vulnerable groups [50-52]. At the same time, many low‑income urban and rural families face food insecurity, low dietary diversity, and heavy reliance on cheap, nutrient‑poor foods, while infant and young child feeding practices (especially complementary feeding quality and frequency) remain suboptimal. The COVID‑19 pandemic and associated economic shocks further undermined household food security and diet quality for low‑income families [53], likely worsening stunting risk even where health and nutrition programs continued. Overall, the country’s strong maternal-child health platforms have not yet been matched by equally strong, equity‑focused action on the food, care, and living environments that determine child growth.

To counter this trend, Malaysia has intensified efforts by identifying effective strategies in its latest national nutrition action plan. Building on these efforts, the National Strategic Plan Book to Combat the Problem of the Double Burden of Malnutrition among Children in Malaysia (2023-2030) has been developed. The Malaysian government’s efforts are now explicitly targeting both undernutrition (including stunting and wasting) and overnutrition in an integrated manner. Through national strategies and guidelines, the government has begun to frame child growth problems not only as undernutrition but also as emerging risks of overweight and obesity, as well as to design programs that respond to this double burden [54].

Myanmar

Despite progress in reducing stunting from over 40% in 1990 to 29.2% in 2016, around 1.4 million children under five remain stunted in Myanmar [55]. The Myanmar National Strategic Plan for Newborn and Child Health Development (2015-2018) aims to achieve specific objectives focused on improving newborn and child health through evidence-based, cost-effective interventions within a Health System Strengthening (HSS) approach [56]. It plans to reduce infant mortality through enhanced home-based and institutional newborn care, and decrease childhood mortality from illnesses like pneumonia, diarrhea, and malaria by improving family practices and community-based care. Strategies include strengthening health systems, improving access to quality services, and boosting demand through community engagement. The plan has set specific coverage targets, including increasing breastfeeding rates, ensuring timely immunization, enhancing treatment coverage for diarrhea and malaria, improving maternal healthcare utilization and delivery practices, increasing the use of adequately iodized salt, and higher rates of vitamin A supplementation among children. These efforts aim to significantly improve the health outcomes of newborns and children, reducing mortality rates and enhancing overall health and well-being.

Additionally, the Myanmar government also devised the Multi-sectoral National Plan of Action on Nutrition (MS-NPAN) 2018/19-2022/23 [55]. Similar to the National Strategic Plan for Newborn and Child Health Development, the MS-NPAN also aims to reduce all forms of malnutrition among mothers, children, and adolescent girls to build a healthier and more productive population. However, the MS-NPAN focuses more on multisectoral collaboration, involving the Ministry of Health and Sports (MoHS), the Ministry of Education (MoE), the Ministry of Agriculture, Livestock and Irrigation (MoALI), and the Ministries of Social Welfare, Relief, and Resettlement (MoSWRR). The MS-NPAN emphasizes collaboration across sectors to deliver coordinated interventions at all levels, aiming to achieve sustainable improvements in nutrition and contribute to a modern, healthy, and prosperous nation.

The MS-NPAN has maternal health objectives that focus on substantial improvements by 2025, including reducing the prevalence of anemia among women of reproductive age from 46.6% in 2015 to 25%, and lowering the rate of low birthweight from 8% to less than 6%. There is also a strong emphasis on increasing the number of women who receive at least four ANC visits and promoting skilled birth attendance, facility-based delivery, and early postnatal care. Additional goals include reducing the maternal mortality ratio and enhancing maternal nutrition, particularly iron and micronutrient status, to further decrease anemia rates. Early and exclusive breastfeeding is a priority, aiming for over 60% for children up to six months. The strategic plan also highlights the importance of improving community recognition of pregnancy danger signs and expanding reproductive health education [56].

By 2025, the MS-NPAN aims to reduce the prevalence of anemia among children under five to 25%. Other health targets include reducing the under-five mortality rate from a baseline of 52 per 1,000 live births in 2015 to 39 per 1,000 by Vision 2030, and decreasing infant mortality from 41 to 30 per 1,000. The prevalence of stunting among children under five is set to be reduced to 21% by 2025, and acute malnutrition (wasting) among this age group is expected to drop to below 5%. The action plan calls for at least 95% coverage of vitamin A supplementation and deworming for children aged 6-59 months, as well as more than 90% coverage for basic immunization and effective disease management. Finally, there is an emphasis on increasing appropriate recognition and referral for danger signs in children, which is particularly relevant for countries such as Myanmar, where public health efforts include raising awareness of at least two critical danger signs in children [56].

While there are no recent national health data to observe the prevalence of stunting of children under five in Myanmar, WHO data estimated the prevalence to be 24.1% in 2022 [24]. Local studies found the prevalence of stunting to be around 27.1% [57] to 28.9% [58]. Reducing stunting in Myanmar requires a coordinated, multisectoral approach that targets both direct and underlying causes. Policies and programs are already implemented in Myanmar, and these efforts involve multi-stakeholders. By scaling up these multi-pronged, evidence-based interventions, Myanmar can make meaningful progress in lowering stunting rates and improving child health and national development.

Philippines

The policies in the Philippines aimed at preventing stunting and improving overall nutrition, which include the Department of Health (DOH) Strategic Framework on Comprehensive Nutrition Implementation Plan 2014-2025, the National Objectives for Health-Philippines 2017-2022, and the Philippine Plan of Action for Nutrition (PPAN). Each of these policies plays a crucial role in addressing malnutrition and enhancing the nutritional status of the population in distinct ways [59-61]. The DOH Strategic Framework on Comprehensive Nutrition Implementation Plan focuses on providing a strategic direction for nutrition programs and interventions in the country. It outlines specific goals, objectives, and strategies to address malnutrition comprehensively. This policy serves as a roadmap for implementing various nutrition initiatives and ensuring that resources are allocated efficiently to combat stunting and other forms of malnutrition [59].

On the other hand, the National Objectives for Health-Philippines 2017-2022 policy set out broader health objectives for the country, including nutrition-related goals. This policy integrates nutrition within the larger framework of public health, emphasizing the interconnectedness of nutrition with overall health outcomes. By including nutrition as a key component of national health objectives, this policy proves the importance of addressing malnutrition as a fundamental aspect of healthcare in the Philippines [60].

The Philippine Plan of Action for Nutrition (PPAN), developed by the National Nutrition Council (NNC), serves as the country’s strategic roadmap to address nutrition challenges and achieve targeted outcomes over six years. The 2023-2028 cycle is the eleventh iteration since 1974 and is closely aligned with major national development initiatives, such as AmBisyon Natin 2040 and the Philippine Development Plan, as well as international commitments and sectoral strategies [61]. The Philippines has achieved significant improvements in child nutrition, particularly in lowering stunting and wasting rates among children under five. Between 2015 and 2021, stunting dropped from 33.4% to 26.7%, while wasting declined from 7.1% to 5.5% during the same period [61].

The PPAN 2023-2028 expands its goals to address a wider range of age groups and incorporates program adaptations for resilience to pandemics and climate change, while maintaining a focus on the first 1,000 days and a life-course approach. Similar to previous versions, it emphasizes multisectoral collaboration, but the latest cycle places stronger leadership and coordination responsibilities on local government units to drive implementation and achieve nutrition targets. The targets for mothers in the PPAN 2023-2028 focus on several crucial outcomes. The plan seeks to reduce the prevalence of nutritionally-at-risk pregnant women and to lower anemia rates in this group from 23% (2018-2019), aiming for a 3.2% reduction each year to bring anemia down to a mild public health concern by 2025, and continue decreasing through 2028. Another priority is to reduce the rate of low birthweight from 14.5% (2017) to 10.2% by 2025, alongside increasing the rate of exclusive breastfeeding for infants in their first six months to 50% by 2028 [61].

For children, the strategic goals include a 50% reduction in under-five stunting by 2030, using the 2013 level of 30.3% as a baseline. There is also a commitment to decrease wasting in children under five from 5.5% (2021-2022) to less than 5% by 2025. These targets reflect an integrated approach to improve maternal and child nutrition outcomes across the country. Interestingly, the PPAN 2023-2028 includes a dedicated section on the enactment of various nutrition-related laws during the previous planning period. All relevant laws, such as those supporting the first 1,000 days program (RA No. 11148 (First 1,000 Days Act)) and expanded maternity leave (the RA 11210 or the 100-day Expanded Maternity Leave Law of 2019, which grants 105 days of paid maternity leave for female workers), have been compiled in one place, making it easier to view the range of nutrition-related policies implemented in the Philippines [61].

The Expanded National Nutrition Survey (ENNS) 2023 reported stunting prevalence for children under five to be 23.6%, and there seemed to be a decreasing trend in stunting rates since 2015 [62]. Over the last five years of implementing the previous PPAN, stakeholders have learned that strong political will and sustained commitment, especially from nutrition-literate leaders at both national and local government levels, are critical to integrating nutrition into the broader development agenda and securing adequate investments in nutrition. Equally important is a genuinely multisectoral approach that mobilizes government agencies, non-governmental organizations (NGOs), and other partners around coordinated actions to address the complex causes of malnutrition, supported by robust communication and advocacy to counter widespread misinformation and raise public awareness of PPAN. Another key lesson is the central role of monitoring, evaluation, and quality data: a well-designed results framework and regular program evaluations are needed to track progress, identify strengths and gaps, generate evidence-based recommendations, and systematically capture and document what local governments are doing so that learning and accountability are strengthened over time [61].

Thailand

According to the Thailand Multiple Indicator Cluster Survey 2022, Survey Findings Report, 12.5% of children under five in Thailand are stunted, while 4.9% are severely stunted [63]. This makes Thailand one of the countries in the SEA region with the lowest prevalence of stunting for children under five. Thailand has implemented several policies and programs to ensure the good health of its population.

Thailand’s 2nd National Reproductive Health Development Policy and Strategy (2017-2026) emphasizes a comprehensive approach to promoting healthy births and child development [64]. By focusing on maternal health, child health and development, family planning, nutrition, adolescent reproductive health, health system strengthening, and the prevention and management of reproductive health issues, Thailand aims to improve reproductive health outcomes and ensure the well-being of mothers and children across the country. A variety of initiatives were promoted, including creating a supportive environment for maternal safety, offering postpartum care, and providing childcare services, all aimed at fostering proper child development and healthy growth through access to educational opportunities. Such movements helped to strengthen current legislation, policies, and initiatives to allow equal access to healthcare services.

Thailand also strongly advocates policies that encourage healthy weight and nutrition for women. Services such as iron and folic acid supplementation to women of reproductive age and the addition of iodine tablets during their pregnancy were made available [65]. Another vital service that was available included providing a standard child growth and development surveillance system [66]. Guidelines from the Maternal and Child Health Handbook could be referred to by the parents and childcare providers to conduct appropriate surveillance on child nutrition, oral health, and development [67]. Another strategy incorporated improving the social welfare system, particularly during the postpartum period, by allowing mothers to take paid ANC without counting it as leave and providing the right to maternity leave. This allowed both the father and mother to take paid leave to care for their children [68].

In addition, Thailand’s nutrition policy has shifted from focusing solely on the first 1,000 days (conception to age two) to covering the entire 2,500-day window (conception to age five) in response to persistent malnutrition among preschoolers and reduced continuity of care after age two [69]. In 2022, the Department of Health initiated a multisectoral collaboration with five ministries, including Interior, Social Development and Human Security, Education, Labor, and Digital Economy and Society, to address these gaps. This integrated, cross-sector approach covers safe pregnancy, workplace breastfeeding spaces, child support grants, nutritious meals in early childhood centers, and oral health promotion. Local and community teams help deliver these services at the grassroots level. The expanded program aligns with Sustainable Development Goals 2 and 3 and the Global Nutrition Targets, embedding comprehensive nutrition care in policies for young children and their families [69].

The Thailand Food Management Action Plan Phase 1 (2023-2027) is a national framework developed to ensure food security, safety, quality nutrition, and sustainability. The plan integrates policies and actions from multiple sectors, including agriculture, health, economic development, and national security, aligning with broader strategies such as the 20-year National Strategy and the 13th National Economic and Social Development Plan. Its overarching vision is for Thailand to become a sustainable source of high-quality, safe, and nutritious food, both for its population and global consumers. In it, Thailand aims to reduce the prevalence of stunting among children under five to below 8% by 2027, decreasing from 11.4% in 2022. The country also seeks to lower the rate of wasting in children under five to under 5% by 2027, down from 5.7% in 2022.

Thailand’s nutrition policies demonstrate a comprehensive and forward-thinking approach focused on the entire population, spanning from preconception and pregnancy through childhood and adulthood. The policies are strongly evidence-based and align with international standards. They prioritize food security, quality, and safety; integrate sustainable agriculture; and promote a multisectoral and life-course approach. Public education, coordination across ministries, and systematic monitoring and evaluation are central to their implementation. Thailand’s framework is well-positioned to continue improving population nutrition and health outcomes.

Timor-Leste

In Timor-Leste, various programs and policies have been put in place to tackle food and nutrition security challenges in the country. One important initiative was the Comoro Declaration signed by all ministers of the Government of Timor-Leste in October 2010. This declaration showed the commitment of various stakeholders to achieve food security for all and to make efforts to eliminate hunger and malnutrition in the country, with a specific goal of reducing the number of malnourished individuals by half by 2015 [70].

Following the Comoro Declaration, the Timor-Leste Strategic Development Plan (SDP) was developed. This plan outlines policies, goals, and implementation of strategies to be carried out in three phases: short-term (2011-2015), medium-term (2016-2020), and long-term (2021-2030). The SDP focuses on four main areas: social capital, infrastructure development, economic development, and institutional framework. It aims to utilize income from oil exports to fund national development efforts [71].

Timor-Leste developed its first National Nutrition Strategy in 2004, introducing evidence-based nutrition interventions and establishing a nationwide program that achieved progress on some key indicators. However, the country continues to experience high rates of malnutrition, with 50.2% stunting, 11% wasting, and 37.7% underweight among children under five. The National Nutrition Strategy (NNS) 2014-2019 provides a stronger multi-sectoral framework for translating national commitments into coordinated actions that address immediate, underlying, and basic causes of malnutrition, aligning nutrition targets with the National Development Plan 2011-2030. Led by the Ministry of Health with technical support from UNICEF, the strategy was developed through a participatory and inclusive process involving all relevant ministries and partners. Its vision is to contribute to sustainable national socioeconomic and human development by improving the quality and productivity of human capital, to improve the nutritional status of the Timorese population and accelerate the reduction of maternal and child undernutrition through nutrition-specific and nutrition-sensitive interventions [72].

For mothers, the NNS 2014-2019 policy aims to reduce anemia among women of reproductive age to below 20%. Efforts are focused on improving maternal nutrition and health care through greater coverage of micronutrient supplementation, iron and folic acid (IFA) provision, deworming, and enhancing access to and use of ANC. The country also seeks to lower the proportion of newborns with low birth weight to under 10%. For children, targets include reducing stunting in those under five to below 40%, underweight rates to less than 30%, wasting to under 10%, and anemia prevalence in children under five to under 40%. Additionally, there are clear mortality goals, such as infant mortality to decrease below 10 per 1,000 live births by 2025, and under-five child mortality to be less than 10 per 1,000 births [72].

In response to the high rate of stunting faced in Timor-Leste, a joint project was implemented in four districts (Aileu, Baucau, Manatuto, and Oecusse). The project focused on improving the health and nutrition status of children under five, pregnant and lactating women, implementing school feeding programs, and establishing a food security and nutrition surveillance system. Strategies included increasing production, availability, and utilization of micronutrient-rich foods through home gardens, small livestock, and aquaculture systems. Community members played a crucial role in identifying and prioritizing nutrition and food security issues, as well as appropriate interventions. However, a mid-term evaluation in 2011 reported no progress toward these interventions, indicating the need for further evaluation and adjustments to ensure the success of these programs in improving nutrition outcomes in Timor-Leste [73]. Despite these efforts, challenges exist in the effective implementation of these programs and policies. The mid-term evaluation in 2011 reported no progress toward the development of interventions in the joint project addressing food insecurity and malnutrition. Delays were also noted in establishing school gardens. Additionally, the underdeveloped health system in Timor-Leste poses challenges in addressing malnutrition, communicable diseases, maternal and child mortality, and limited access to healthcare facilities [74,75].

WHO data estimated stunting of children under five in Timor-Leste to be 45.1% in 2022 [24]. This shows that further actions are needed. Timor‑Leste already has comprehensive nutrition policies; further progress on stunting will depend on implementation quality, geographic prioritization, and system‑level enablers rather than new policy documents. The country should strengthen delivery and coverage of existing high‑impact interventions in the first 1,000 days, with a specific focus on high‑burden rural municipalities, and ensure that health, agriculture, WASH, education, and social protection programs are co‑located so households receive an integrated package of support. Additionally, empowering the national stunting‑reduction coordination mechanism with clear authority, adequate and predictable financing, and a robust monitoring and accountability framework can help close the gap between policy intent and on‑the‑ground impact.

Vietnam

Four decades ago, Vietnam faced severe food insecurity and famine, with stunting rates at 61.3% in 1988 and 61.5% in 1995. Concerns over malnutrition prompted the introduction of the National Plan of Action for Nutrition (NPAN) from 1995-2000, followed by subsequent strategies spanning 2001-2010 and 2011-2020. Over the past two decades, by emphasizing malnutrition, including stunting, in their nutrition policies, Vietnam has significantly improved the health of children under five years old [76]. Vietnam’s recent policy document, the approved National Nutrition Strategy (NNS) for 2021-2030 with a vision to 2040, includes plans to revise, enhance, and finalize legal provisions on nutrition [77]. The strategy prioritizes nutrition interventions in disadvantaged areas, remote regions, ethnic minority communities, mountainous regions, and islands.

For mothers, the NNS 2021-2030 aims to reduce anemia among women of reproductive age to less than 8% nationally and below 15% for women in mountain areas and ethnic minorities by 2030. Additional objectives include reducing the proportion of newborns with low birth weight to under 5% by 2025 and maintaining low levels by 2030, lowering anemia in pregnant women nationally to below 15% (and below 25% in high-risk groups), and improving women’s dietary diversity and micronutrient status.​

For children, the NNS targets include reducing stunting in those under five to less than 15% nationally, with a goal of under 25% in mountain and ethnic minority populations by 2030. Wasting is aimed to be reduced to under 5%, underweight rates to less than 10%, and exclusive breastfeeding in the first six months is aimed to rise to 55% by 2030. The prevalence of anemia among children under five is expected to drop to 10% nationwide (with 20% as the goal for mountainous/ethnic minority children). Efforts also prioritize reducing vitamin A deficiency in children under five to less than 4% nationally and 10% in high-risk areas.​ These specific, equity-focused goals reflect Vietnam’s commitment to addressing persistent regional disparities in nutrition outcomes and supporting the health of its most vulnerable populations.

The NNS 2021-2030 also proposes the development of policies and financial mechanisms, such as health insurance coverage for nutrition-related activities in healthcare facilities and schools. Vietnam aims to reduce childhood stunting to below 17% by 2025 and below 15% by 2030, emphasizing these targets as crucial socioeconomic milestones in its development goals. The policy also aims to strengthen intersectoral collaboration and mobilize social organizations and business communities to participate in nutrition activities [77].

Through the implementation of strategies in the NNS, Vietnam’s nutrition strategy aims to strengthen communication and education by tailoring messages for different regions and target groups, improving counseling, and fostering collaboration between schools, families, and communities to prevent stunting. Efforts include expanding nutrition messaging through television, radio, social media, and digital platforms while developing a network of trained nutritionists and village health workers with standardized curricula and enhanced technical skills. The plan emphasizes better meal quality, food security, and agriculture models, alongside essential nutrition interventions during the first 1,000 days of life, covering maternal care, exclusive breastfeeding, complementary feeding, growth monitoring, malnutrition management, and micronutrient supplementation and fortification. To ensure sustainability, Vietnam integrates nutrition services with health, education, poverty reduction, and social protection programs, targeting vulnerable groups such as ethnic minorities and those in rural areas [77].

The General Nutrition Survey in 2019 reported that 19.6% of children under five in Vietnam were stunted [78], while the most recent WHO data estimated 19.3% of children under five to be stunted in 2022 [24]. Vietnam is making steady progress in reducing child stunting by implementing comprehensive nutrition policies and programs that focus on equitable outcomes for vulnerable groups. The government has set a target to lower stunting among children under five to below 15% nationally and below 25% among those in the mountains and ethnic minorities by 2030. These efforts include multisectoral strategies that address both maternal health and child nutrition, promote exclusive breastfeeding, improve dietary diversity, and deliver micronutrient interventions, with a particular emphasis on reaching disadvantaged communities.

Overview of policies and programs to reduce stunting in Southeast Asian countries

Mapping Policies to the UNICEF Conceptual Framework

The UNICEF Conceptual Framework on Maternal and Child Nutrition (2021) provides a holistic, layered understanding of the determinants of nutrition for mothers and children [11]. It distinguishes between enabling determinants (such as governance, resources, and social norms), underlying determinants (including the availability of nutritious food, age-appropriate feeding and dietary practices, and access to nutrition, health, and sanitation services), and immediate determinants, which focus on the quality of diets and care received by women and children. The framework makes clear that positive nutrition outcomes depend on political, financial, social, and cultural conditions, as well as adequate services, good feeding practices, and healthy living environments. Improved nutrition leads to better health, cognitive development, school readiness, productivity, and overall societal prosperity, emphasizing the interconnectedness of individual, household, and systemic drivers of nutrition.

Enabling Determinants

Enabling determinants include governance, resources, and norms. Good governance encompasses political, financial, social, public, as well as private sector measures. Resources include sufficient resources, such as environmental, financial, social, and human resources. Norms comprise positive social and cultural norms as practices that would benefit children’s and women’s rights to nutrition.

In terms of governance, all SEA countries have policies that stress the importance of multisectoral collaborations to ensure their goals can be achieved. These include inter-ministerial cooperations, as well as partnerships with the private sector, academia, civil society organizations (CSOs), and NGOs.

When it comes to resources, most countries, such as Malaysia, Vietnam, Thailand, and Myanmar, now have high-level, multisectoral coordinating bodies or committees to drive nutrition policy. However, implementation capacity varies. High-income or upper-middle-income countries (Malaysia, Thailand) tend to have more stable government financing for nutrition actions, while lower-income nations (Timor-Leste, Lao PDR, Cambodia, Myanmar) are more reliant on donor support or special external funding mechanisms. In these latter settings, budget lines for nutrition exist, but often only partial funding is available, resulting in bottlenecks and “unfunded plans.”

Decentralization of responsibility is common (e.g., local government program implementation in the Philippines and Vietnam), but challenges in sub-national planning and delivery, due to staff shortages, poor vertical integration, or lack of specialized workforce, create gaps between policy and practice (as mentioned in the Indonesia Stranas, Lao PDR, and Myanmar policies). National plans usually integrate nutrition into broader socio-economic or health strategies, yet “nutrition-specific” budget tracking and monitoring are often insufficiently developed, limiting accountability. This is further discussed below.

For instance, Indonesia’s national strategy for accelerating stunting reduction faces several critical funding challenges. Budget allocations at all administrative levels, national, provincial, district, and village, are structured in a generalized manner, making it difficult to direct resources to specific target villages where interventions are most needed. Fiscal capacity in regional and village budgets remains limited, constraining the ability to finance prioritized nutrition programs at scale [30]. Moreover, government funding across levels, as well as from legal alternative sources, is often channeled more toward physical infrastructure than to human development, leaving policy-driven stunting reduction efforts under-resourced. Additionally, many regional governments lack the skills and systems to accurately estimate their medium-term funding requirements and struggle to mobilize and sustain the necessary investments for program expansion, which further hinders progress in scaling up stunting interventions [30].

A review of Laos’ previous NPAN highlighted several financing and implementation gaps, emphasizing the need for realistic budgeting with deeper Ministry of Finance involvement and full integration of nutrition costs into sectoral budgets. The new NPAN 2021-2025 plans to conduct a new costing exercise in response. Nutrition programming in Laos remains overwhelmingly dependent on external donors, with over 90% of funds from official development assistance in recent years [37]. This reliance means that many local agencies and NGOs have minimal domestic resources for nutrition, jeopardizing program continuity when donor funding ends, especially for multisectoral activities involving WASH, agriculture, and nutrition education, which are difficult to sustain due to siloed government funding. Persistent challenges also include insufficient intersectoral integration, capacity gaps, adaptation to new funding mechanisms, impacts from COVID-19, and weak routine monitoring and coordination. The new NPAN directly addresses these issues by focusing on better integration, workforce capacity, and more robust monitoring and reporting systems [37].

Myanmar’s MS-NPAN 2018/19-2022/23 aims to use refined indicators and leverage existing sector-specific information systems for data collection and performance tracking, provided these systems are reliable. However, several significant constraints hinder effective monitoring and evaluation. These include a shortage of skilled personnel, inadequate infrastructure and technological tools, and insufficient budget prioritization for monitoring and evaluation [55]. Routine reporting on program activities is uncommon, especially outside development partner-assisted projects, and there is limited capacity at the implementation level to analyze and use data for decision-making. Data verification is often weak due to funding constraints, and there is a general lack of awareness about the importance of both data reliability and monitoring and evaluation. Even when reports are submitted, feedback and actionable analysis from higher levels are rare. Moreover, information from NGO-led projects is not consistently integrated into the government’s data system, resulting in further fragmentation and missed opportunities for program improvement [55].

Maternity leave is another example of enabling determinants, as these are policies enacted at the national level to encourage mothers to stay home post-partum, which hopefully could increase exclusive breastfeeding. As shown in Table 1, not all SEA countries have specified maternity leave in their policy documents, but some do. Brunei, Cambodia, Malaysia, Myanmar, the Philippines, and Thailand were some that did mention about paid maternity leave. Some policy documents even stated plans for paternity leave. Certain policy documents, such as those of Indonesia and Vietna,m did not mention maternity leave, but the policy was enacted through other laws and regulations. Vietnam, for instance, has one of the longest paid maternity leave policies of six months (in comparison, Brunei has 105 days and Malaysia suggested extending the leave to 98 days) [79]. However, whether this type of policy is effective remains to be seen, as there may be variability in the execution at the grassroots level [80].

**Table 1:** A summary of some findings from policy documents reviewed. Note: These indicators are based on the reviewed documents. If the indicator is not available in the table, it may be present in other regulations or laws not reviewed here. ^1^: Some countries may not have the most recent national health survey data; thus, other references were used. ANC: antenatal care; BFHI: breastfeeding-friendly hospital initiative; EBF: exclusive breastfeeding; IFA: iron and folic acid supplementation; LBW: low birth weight; WASH: water, sanitation, and hygiene

Country	Documents reviewed	Maternal anemia	LBW	EBF	ANC	Maternal supplements	Types of supplements	Stunting	Wasting	Underweight	Immunization	First 1,000 days	BFHI	Maternity leave	WASH	Stunting target (%)	Current stunting prevalence (%)	Stunting indicator reference(s) ^1^
Brunei	2014–2020 (National Strategy for Maternal, Infant & Young Child Nutrition (MIYCN)) [21]. Maternity Leave Regulation 2011 [22]	Yes	Yes	Yes	Yes	Yes	Prophylaxis folic acid, prophylaxis ferrous fumarate	Yes	Yes	-	Yes	-	Yes	Yes	-	≈12% by 2025	10.9%	WHO Data [24]
Cambodia	Fast Track Road Map for Improving Nutrition 2014-2020 [26]. 2nd National Strategy for Food Security and Nutrition 2019-2023 [27]. National Guidelines for the Implementation of Baby-Friendly Hospital Initiative in Cambodia [82]	Yes	Yes	Yes	Yes	Yes	Daily IFA for pregnant mothers	Yes	Yes	Yes	Yes	Yes	Yes	Yes	Yes	25% in 2023	21.9%	Cambodian Demographic and Health Survey (CDHS)-2021/2022 [29]
Indonesia	National Strategy To Accelerate Stunting Reduction (Stranas Stunting) 2018-2024 [30]. National Medium-Term Development Plan/RPJMN [32]	Yes	Yes	Yes	Yes	Yes	Iron supplement tablets, calcium supplements for pregnant mothers, vitamin A supplement capsules, multiple micronutrient supplements for lactating mothers and children (0-23 months)	Yes	Yes	Yes	Yes	Yes	Yes	Yes	Yes	14%	19.8%	Indonesian Nutritional Status Survey (SSGI) 2024 [36]
Laos	National Plan of Action on Nutrition (NPAN) 2021-2025 [37]	Yes	Yes	Yes	Yes	Yes	Vitamin A supplementation for children under 5; IFA tablets for pregnant women	Yes	Yes	Yes	Yes	Yes	Yes	Yes	Yes	26% by 2025	32.8%	Lao Social Indicator Survey III, 2023, Survey [40]
Malaysia	National Plan of Action for Nutrition of Malaysia III (NPANM III), 2016-2025 [44]. Handbook: Mid-Term Review of The National Plan of Action For Nutrition of Malaysia (NPANM) III, 2016-2025 [44]. Manual for the First 1000 Days of a Child’s Life: What Parents and Guardians Need to Know [48]	Yes	Yes	Yes	Yes	Yes	IFA for pregnant mothers	Yes	Yes	Yes	-	Yes	Yes	Yes	-	11.8%	21.2%	National Health and Morbidity Survey (2022) [49]
Myanmar	National Strategic Plan for Newborn and Child Health Development (2015-2018) [56]. Multi-sectoral National Plan of Action on Nutrition (MS-NPAN) 2018/19-2022/23 [55]	Yes	Yes	Yes	Yes	Yes	Multiple micronutrient or iron tablet supplementation for pregnant women; vitamin B1 for pregnant and lactating women	Yes	Yes	-	Yes	-	Yes	Yes	Yes	21% by 2025	24.1%, 27.1%, 28.9%	[24], [57], [58]
Philippines	The Philippine Plan of Action for Nutrition (PPAN) 2023-2028 [61]	Yes	Yes	Yes	Yes	Yes	Vitamin A capsules for pregnant and lactating women and young children; IFA for pregnant women; Iodized oil for Pregnant and lactating women in areas endemic to iodine deficiency disorders with poor access to adequately iodized salt	Yes	Yes	-	Yes	Yes	Yes	Yes	Yes	15.2% by 2030	23.6%	Expanded National Nutrition Survey (ENNS) 2023 [62]
Thailand	Thailand Food Management Action Plan Phase 1 (2023-2027) [83]. The 2nd National Reproductive Health Development Policy and Strategy (2017-2026): Promotion of Healthy Birth and Child Development [64]. First 2500 days of life program [69]	Yes	Yes	Yes	Yes	Yes	IFA and iodine supplements during pregnancy.	Yes	Yes	-	Yes	Yes	Yes	Yes	-	<8% by 2027	12.5%	Thailand Multiple Indicator Cluster Survey 2022 [63]
Timor-Leste	Timor-Leste Strategic Development Plan 2011 – 2030 [71]. Timor-Leste National Nutrition Strategy 2014-2019 [72]	Yes	Yes	Yes	Yes	Yes	Vitamin A, IFA for pregnant and lactating mothers	Yes	Yes	Yes	Yes	Yes	Yes	-	Yes	<40%	45.1%	WHO data set [24]
Vietnam	Vietnam National Nutrition Strategy (2021-2030 with vision to 2040) [77]	Yes	Yes	Yes	-	Yes	Multi-micronutrient supplementation; IFA to pregnant women	Yes	Yes	Yes	-	Yes	Yes	-	Yes	<15% by 2030	19.3%	WHO data set [24]

Taken together, these findings show that while SEA countries have established governance structures, multisectoral policies, and high-level coordinating bodies that recognize nutrition as a priority, weak and uneven resourcing, fragmented financing, and limited monitoring and evaluation capacity significantly undermine implementation on the ground. Reliance on external donors, inadequate and poorly targeted budget allocations, staffing and skills gaps at sub-national levels, and weak data systems collectively create a disconnect between ambitious plans and the delivery of effective, sustained nutrition actions for women and children, highlighting the need to strengthen domestic financing, implementation capacity, and accountability mechanisms across all levels of government.

Underlying Determinants

Underlying determinants include food, practices, and services. With respect to underlying determinants, all countries feature a mix of nutrition-specific and nutrition-sensitive interventions, but their scope and integration differ. Stunting, wasting, anemia, and exclusive breastfeeding are prioritized everywhere, yet only certain countries, such as Myanmar and Vietnam, set explicit targets for equity to reach vulnerable groups such as ethnic minorities or those in remote areas.

In Myanmar, for instance, the MS NPAN 2018/19-2022/23 is tailored to reflect the unique conditions and needs of each of Myanmar’s 15 states and regions. Stunting is considerably more prevalent in rural Myanmar (31.6%) than in urban areas (21.0%), with the highest state-level rates seen in Chin (41.0%), Kayah (39.7%), and Rakhine (37.5%). The largest numbers of stunted children are found in Shan, Ayeyawaddy, and Mandalay regions [55]. In Vietnam, the NNS 2021-2030 specific targets are set for vulnerable groups, such as ethnic minorities and those in the mountain areas [77].

WASH are addressed in the national policies of some SEA countries, but not all. Brunei, Thailand, and Malaysia, for instance, did not stress the WASH component in their policies. Whereas in other countries, WASH is deemed important to improve the livelihood of the population, such as in Cambodia, Laos, Myanmar, and the Philippines.

Additionally, effective cross-sector convergence remains challenging. Laos, for example, has well-designed policies on paper but faces struggles with weak local delivery and sectoral silos. Indonesia and the Philippines have incorporated broader social protection tools such as conditional cash transfers and school-based nutrition activities, yet sustaining these programs and demonstrating consistent results remain concerns. Real-time monitoring, systematic learning, and strong integration between nutrition-specific and nutrition-sensitive interventions are generally lacking, limiting the full realization of outcomes.

Immediate Determinants

Immediate determinants include diets and care. As can be seen in Table 1, which summarizes some findings from policy documents reviewed, all SEA countries have similar goals and approaches to ensure the maternal and child well-being of their populations. For instance, most policies emphasize the first 1,000 days and target high-impact interventions: ANC, micronutrient supplementation, breastfeeding support, and treatment for wasting and infections. However, the delivery of these services varies. Coverage of basic services (e.g., ANC, immunization, and community-based nutrition promotion) is higher and more consistent in countries with more robust public health infrastructures and investment in health workforce (Malaysia, Vietnam, Thailand), but much lower in fragile or resource-limited states (Timor-Leste, Myanmar, Cambodia). Human resource shortages, weak community health platforms, and logistics/supply chain challenges frequently constrain program effectiveness. These are especially clear in Timor-Leste and Laos, and these limitations are acknowledged in strategy documents.

Incorporating Stunting as a National Indicator

All of the 10 SEA countries reviewed have embraced evidence-based recommendations outlined in the WHO Stunting Policy Brief (2014) to effectively combat stunting [19]. Their strategies integrate a blend of nutrition-specific and nutrition-sensitive approaches, which can change the determinants of malnutrition to enhance growth and development [81]. Recognizing its critical impact, each country has incorporated stunting as a nutritional indicator in its national policies. This ensures that specific targets align with global nutrition goals and are regularly monitored, enabling timely adjustments to achieve these targets effectively. Also, it is vital for monitoring the Sustainable Development Goals (SDGs) and worldwide reporting, assuring comparable and reliable data. Indonesia has implemented targeted policies such as the National Strategy to Accelerate Stunting Reduction (StraNas Stunting) to address stunting, ensuring national attention to the issue [30]. Likewise, the Lao DPR government recently carried out the Lao Social Indicator Survey III (LSIS III), which was part of the Global Multiple Indicator Cluster Survey (MICS) Program and was carried out by the Lao Statistics Bureau (LSB) in partnership with the Ministry of Health and the Ministry of Education and Sport. The 2,500 first days of life policy implemented in Thailand also aligns closely with SDG2 and 3, as well as Global Nutrition Targets.

Thus, it is clear that all SEA countries have incorporated stunting as a national indicator, and goals set in policies of each country align closely with international standards. This is important for ensuring common standards for assessing stunting, enabling evaluations and comparisons between regions, nations, and research areas [40].

However, monitoring and evaluation is still an ongoing issue in several countries. Although policies and programs have been devised, the effectiveness and impact of each policy and the implemented programs need to be continuously tracked and revised as needed. This review found that not all SEA countries have national health surveys, which could impede the monitoring and evaluation process. Countries such as Brunei, Myanmar, Vietnam, and Timor-Leste lack more recent national public health data, or are not accessible for an international audience (for instance, reports are not available in English). Instead, estimates of stunting indicators had to be done based on the WHO dataset estimates. Prioritizing nutrition health surveys requires strong political will and financial support from the government, and it is essential to ensure the policies and programs are executed based on evidence.

Challenges

Bottleneck analysis further clarifies the factors underlying divergent policy impact across the region. Stable domestic financing supports sustained program investments in wealthier countries, but elsewhere, dependence on external funding exposes programs to instability and disrupts continuity. Where the nutrition workforce is not integrated as a core function within health or other sectors, or where there are major gaps in district and community-level capacity, service coverage suffers even when policies are ambitious. Monitoring and evaluation systems are frequently under-resourced or fragmented, with only a subset of countries maintaining strong national nutrition surveillance and feedback loops. Behavior change and social mobilization, though highlighted in all strategies, face significant challenges in achieving widespread and sustained impact, particularly in hard-to-reach communities.

Opportunities

Countries that maintain sustained domestic investment in nutrition, coupled with strong health infrastructure and effective local governance, tend to achieve better outcomes in reducing core malnutrition indicators and narrowing equity gaps. This pattern is evident in places such as Thailand and Vietnam, where robust monitoring and evaluation systems and integrated social protection measures are also in place. In contrast, countries where funding is unpredictable, workforce capacity remains thin, or monitoring relies heavily on donor-driven and siloed processes, such as Timor-Leste and Laos, find it more difficult to achieve and maintain rapid improvements, especially during shocks or when programs are scaled up. Nutrition-sensitive strategies, including school feeding, cash grants, WASH, and nutrition-friendly agriculture, show considerable promise, but they are only effective when they are well-coordinated and embedded within broader poverty reduction and social protection frameworks.

Moreover, regular monitoring, systematic learning, and the presence of structured mechanisms for feedback and course correction are distinguishing features of countries that demonstrate sustained success. In Vietnam, for instance, the NNS 2021-2030 set specific targets for ethnic minorities and populations living in remote mountainous regions, and these targets differ from the national targets of the main populations. This proved that the Vietnamese government recognized urban-rural and minority disparities. By setting separate targets, intervention efforts are more focused and are tailored to specific conditions of the populations. This could potentially be implemented in other nations that struggle with persistent stunting issues, such as Malaysia, which has shown disparities in health between urban-rural and remote populations.

## Conclusions

In summary, this review showed that all SEA countries have set ambitious goals in their policies, which align with international standards, and several programs have been implemented to reach these goals. Some countries in the region have made significant strides to reduce the prevalence of stunting in children under five, such as Brunei, Cambodia, Thailand, and Vietnam. There are also countries, such as Indonesia and Myanmar, that have made remarkable progress, even if the stunting prevalence did not quite achieve the targets set in national policies. Meanwhile, some countries did not quite reach their targeted goals, such as Laos, Malaysia, the Philippines, and Timor-Leste. In these countries, existing nutrition policies may only focus on nutrition-specific and nutrition-sensitive interventions, but systematic economic issues, income and wealth disparities, as well as regional differences between urban and rural areas, may not be adequately addressed by nutrition policies alone. In these instances, more targeted interventions such as cash transfers/assistance (like what was done in Cambodia, Indonesia, and Myanmar), or specifically targeting minority and/or vulnerable groups (like what was done in Myanmar and Vietnam), may be more effective. WASH interventions may need to be intensified in selected countries, as this may be a big issue that could affect children’s development. A standardized system for monitoring child growth and development can also be implemented (as was done in Thailand), along with nutrition education to the population regarding child growth. This could potentially empower parents to keep track of their child’s growth and seek help if there are any concerns. SEA countries share a broad recognition of nutrition’s multisectoral, social-determinant roots, and most have established comprehensive strategies. Policy bottlenecks persist where there are financing deficits, staffing and structural challenges, or where delivery and data systems are fragmented. Greater progress is achieved when countries embed nutrition within broader health and social development systems, invest in frontline staff, establish robust monitoring and evaluation, and systematically address vulnerable populations. Applying the UNICEF conceptual framework allows for clearer identification of challenges, facilitating more targeted improvements across the region. Ultimately, this narrative review provides a consolidated understanding of stunting trends across Southeast Asia, allowing policymakers and practitioners to identify common gaps and effective strategies. By synthesizing regional evidence, the review supports more coordinated, context-specific actions to accelerate progress in reducing stunting.
